# Mapping Cortical Responses to Somatosensory Stimuli in Human Infants with Simultaneous Near-Infrared Spectroscopy and Event-Related Potential Recording^1^,^2^,^3^

**DOI:** 10.1523/ENEURO.0026-16.2016

**Published:** 2016-05-13

**Authors:** Madeleine Verriotis, Lorenzo Fabrizi, Amy Lee, Robert J. Cooper, Maria Fitzgerald, Judith Meek

**Affiliations:** 1 Department of Neuroscience, Physiology and Pharmacology, University College London, London WC1E 6BT, United Kingdom; 2 Department of Medical Physics and Biomedical Engineering, University College London, London WC1E 6BT, United Kingdom; 3 Elizabeth Garrett Anderson Obstetric Wing, University College Hospital, University College London Hospitals, London, WC1E 6DB, United Kingdom

**Keywords:** EEG, hemodynamic, multimodal, neurovascular coupling, noxious, pain

## Abstract

Near-infrared spectroscopy (NIRS) and electroencephalography (EEG) have recently provided fundamental new information about how the newborn brain processes innocuous and noxious somatosensory information. However, results derived independently from these two techniques are not entirely consistent, raising questions about the relationship between hemodynamic and electrophysiological responses in the study of touch and pain processing in the newborn. To address this, we have recorded NIRS and EEG responses simultaneously for the first time in the human infant following noxious (time-locked clinically required heel lances) and innocuous tactile cutaneous stimulation in 30 newborn infants. The results show that both techniques can be used to record quantifiable and distinct innocuous and noxious evoked activity at a group level in the newborn cortex. Noxious stimulation elicits a peak hemodynamic response that is 10-fold larger than that elicited by an innocuous stimulus (HbO_2_: 2.0 vs 0.3 µm) and a distinct nociceptive-specific N3P3 waveform in electrophysiological recordings. However, a novel single-trial analysis revealed that hemodynamic and electrophysiological responses do not always co-occur at an individual level, although when they do (64% of noxious test occasions), they are significantly correlated in magnitude. These data show that, while hemodynamic and electrophysiological touch and pain brain activity in newborn infants are comparable in group analyses, important individual differences remain. These data indicate that integrated and multimodal brain monitoring is required to understand central touch and pain processing in the newborn.

## Significance Statement

Processing of touch and pain in the developing newborn brain can be studied using near-infrared spectroscopy (NIRS) and electroencephalography (EEG). However, the relationship between hemodynamic and electrophysiological responses to somatosensory stimuli in the newborn is not known. We recorded NIRS and EEG responses simultaneously, and found that hemodynamic and electrophysiological touch and pain brain activity in newborn infants are comparable in group analyses; however, single-trial analysis revealed that these responses do not always co-occur in individual trials. This important variability suggests that integrated and multimodal brain monitoring is required to understand central touch and pain processing in the newborn.

## Introduction

Newborn infants are exposed to a wide range of cutaneous sensory stimuli in the first few days of life. Most of these are innocuous mechanical stimuli, such as touch or light pressure, although noxious skin-breaking procedures are also performed in neonates requiring hospital care. Little is known about how the developing newborn cortex processes these stimuli, but cortical activation by noxious and innocuous mechanical stimulation has been recorded at the cotside using near-infrared spectroscopy (NIRS; Bartocci et al., 2006; Slater et al., 2006) and electroencephalography (EEG; Slater et al., 2010a; Fabrizi et al., 2011). Although these recordings offer great potential for investigating the postnatal development of human cortical somatosensory and pain networks, the data from these techniques have not been entirely consistent, raising the question of whether the two techniques are measuring the same integrated cortical activity following cutaneous noxious and innocuous stimulation.

Studies using NIRS have reported a clear hemodynamic response over the contralateral primary somatosensory cortex (SI) following noxious heel lance and noxious venipuncture in newborn infants (Bartocci et al., 2006; Slater et al., 2006). The hemodynamic response to heel lance was observed in single trials, was clear from 25 weeks gestational age (GA), and increased with age. It was also smaller in sleeping infants compared to awake infants at the same age. By contrast, no response was detected following innocuous mechanical stimulation using von Frey hairs at intensities sufficient to elicit visible foot withdrawal (Slater et al., 2006), although a small response was reported following skin disinfection (Bartocci et al., 2006).

Studies using EEG have reported clear event-related potentials (ERPs) at the vertex following both noxious and innocuous stimulation (Slater et al., 2010a,b; Fabrizi et al., 2011). The EEG response time locked to a noxious heel lance consists of two ERPs, the last of which is nociceptive specific (N3P3; N150-P260 and N420-P560), while the response following innocuous tactile stimulation consists of only the first ERP (N2P2). It is possible that these waveforms are preceded by an earlier somatosensory evoked potential (also referred to as the N1P1; for review, see Vanhatalo and Lauronen, 2006), but this has not been reported yet. Both ERPs can be observed in single trials, but, unlike hemodynamic responses, they only begin to appear reliably from ∼37 weeks of age (Fabrizi et al., 2011). Furthermore, in contrast to the hemodynamic response, the N2P2 waveform is larger in sleeping infants, while the nociceptive-specific waveform is not dependent on sleep state (Slater et al., 2010a).

Some discrepancies between sensory evoked neural and hemodynamic responses in infants may be due to methodological differences; for instance, the criteria for detecting a tactile hemodynamic response may have been too stringent in previous studies, given the likely low signal-to-noise ratio (Slater et al., 2006). Likewise, the presence of a hemodynamic response in preterm infants in which ERPs are rarely observed could be related to different patterns of EEG activity in this age group, such as delta brushes (Fabrizi et al., 2011). Nevertheless, the differences raise the possibility that the two techniques are not measuring (either directly or indirectly) the same integrated somatosensory cortical activity.

To address this issue, we have recorded NIRS and EEG simultaneously in individual healthy term babies. Hemodynamic activity was recorded from the contralateral SI as it contributes, at least in part, to the generation of the adult noxious event-related potential (Valentini et al., 2012; Hu et al., 2014), and is therefore likely to contribute to the infant ERP. We predicted that, with improved recording and analysis techniques, cortical responses to both innocuous and noxious mechanical skin stimulation could be quantified in neonates using both NIRS and EEG. Furthermore, we predicted that this would also be true in single trials so that evoked hemodynamic and electrophysiological activity, when recorded simultaneously in individual trials, would always co-occur and be correlated. To test these hypotheses, we developed a novel goodness-of-fit (GOF) analysis for the evoked hemodynamic activity that allowed comparison and correlation with the principal component analysis (PCA) of evoked EEG activity.

## Materials and Methods

### Participants

Thirty-six healthy term infants were recruited from the postnatal ward and special care baby unit at the Elizabeth Garrett Anderson Obstetric Wing, University College Hospital (UCH). Infants were not eligible for inclusion in the study if they were born in poor condition, had congenital malformations, or were receiving analgesics at the time of study. All infants and their mothers were well at the time of the study. Infant demographics and clinical details are shown in Table 1.

**Table 1.** Demographic characteristics of participating infants

**Demographic information**	
No. infants	36
Age at birth (weeks)	39.0 (36.3–42.0)
Age at study (weeks)	39.2 (36.6–43.3)
Postnatal age at study (d)	2 (0–16)
Female infants	15/36
Infants receiving right heel stimulation	20/36
Weight at birth (g)	3257 (1920–4750)
Cesarean deliveries	18/36

Data are shown as the median (range) or as *n*/*N* and refer to number of infants, unless otherwise indicated.

Ethical approval for this study was given by the UCH ethics committee. Informed written parental consent was obtained before each study. The study conformed to the standards set by the Declaration of Helsinki.

### Experimental protocol

Each infant received at least one of the following three types of stimuli: noxious, control, or tactile.

#### Noxious stimulus

Twenty-one infants were studied during a clinically required routine heel lance for the purpose of obtaining blood samples. No heel lances were performed solely for the purpose of the study, and all samples were obtained by a nurse or doctor using a lancet (Tenderfoot, ELITech UK Ltd). The heel area was cleaned at least 30 s prior to the lance, and the heel was not squeezed until at least 30 s after the lance to enable the assessment of cortical responses to the lance stimulus alone.

#### Control stimulus

To control for the tactile (due to the placement of the lancet onto the heel) and auditory (due to the audible “click” that is produced when the spring-loaded blade is released) aspects of the lance, all infants also received a control stimulus at least 2 min prior to the heel lance. This involved placing the lancet onto the heel in a 90° rotated position compared with the lance, so that when it was triggered the blade was released away from the foot. As with the noxious stimulus, care was taken to minimize other stimulation during the 30 s prior to and following the control stimulus.

#### Tactile stimulus

A separate group of 15 infants, and 1 of the infants that were studied during lance, received tactile stimulation, which consisted of a gentle tap to the heel using a custom-made tendon hammer. All infants received 10 taps with a mean interstimulus interval of 72 s (SD, 16 s), except for three infants who received fewer taps due to limited time. For the infant who received tactile stimulation in addition to the lance, tactile stimulation was given prior to the control stimulus, and the heel was lanced last.

All stimuli were time locked to the EEG and NIRS recordings using a movement transducer attached to the lancet or tendon hammer (Worley et al., 2012).

#### Infant well-being

Throughout the experiment, care was taken to ensure the well-being and comfort of the babies and their families. Following hospital policy, comfort care was used rather than sucrose. Parents were always present during the study and were able to hold their baby if they wished. Babies were fed on demand. EEG and NIRS sensors were placed gently on the head by a trained clinical physiologist; handling of the babies was otherwise kept to a minimum throughout the experiment, and nearly all infants were still asleep prior to the noxious heel lance (see EEG recording). Parents were informed that they could stop the experiment at any time.

### Multimodal recording

Cortical activity was measured with simultaneous NIRS and EEG recordings in 30 infants. In the remaining six infants only NIRS recordings were undertaken due to limited time.

#### NIRS recording

Hemodynamic activity was recorded at a sampling rate of 5 Hz using the NIRO-200NX (Hamamatsu Photonics K.K.), which uses three wavelengths (735, 810, and 850 nm, with an output power of <2 mW). Light absorption was converted into changes in [HbO_2_] and [HHb] at the outset using the modified Beer-Lambert law with a differential path-length factor of 4.39 (Wyatt et al., 1990).

**Figure 1.**
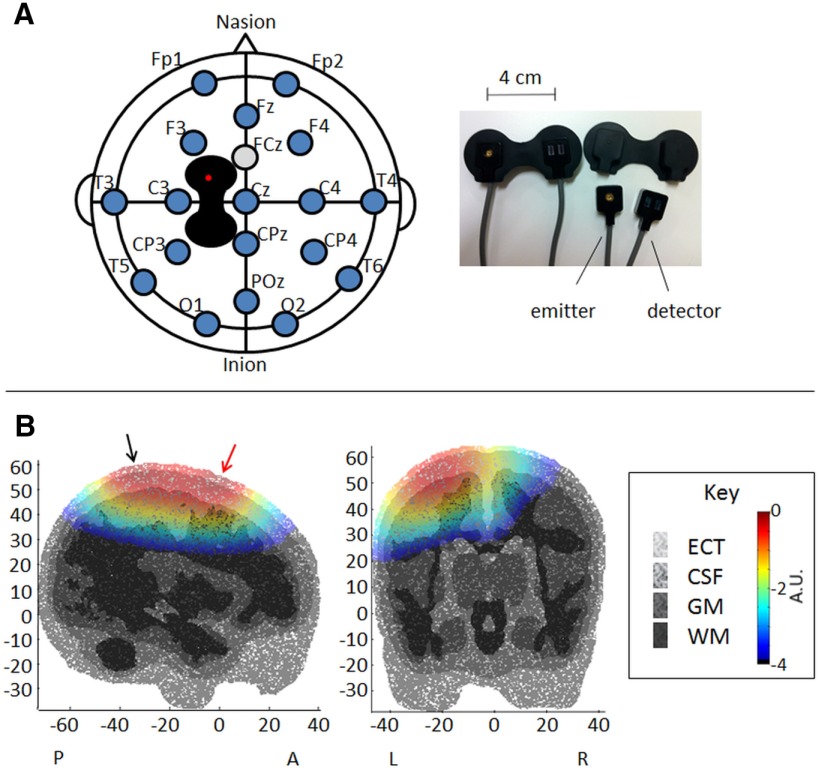
***A***, ***B***, NIRS optode and EEG electrode locations (***A***) and NIRS sensitivity maps (***B***). ***A***, NIRS optode (black) and EEG electrode (blue circles) locations are presented on a schematic of the top view of the head (left). The EEG reference electrode was placed at FCz (gray circle). The NIRS emitter and detector were placed in a holder (pictured right) at a fixed distance of 4 cm, with the emitter toward the front of the head (red dot in schematic). ***B***, The sensitivity map of the optodes is shown at C1 (halfway between Cz and C3) in 2-mm-thick sagittal (left) and coronal (right) slices taken from a head model of 40-week-old infants. The scale bar indicates the log of the normalized sensitivity (in arbitrary units). A high sensitivity indicates that many photons pass through the given region on their way to a detector. Red and black arrows indicate the emitter and detector locations. A, Anterior; P, posterior; L, left; R, right; ECT, extracerebral tissue; CSF, cerebrospinal fluid; GM, gray matter; WM, white matter.

Although near-infrared light travels diffusely through tissue, the sensitivity distribution associated with a given emitter–detector pair has a well described shape that resembles a banana, with narrow ends at the emitter and detector and the highest depth sensitivity at the midpoint between the optodes (Arridge, 1995; Lloyd-Fox et al., 2010a; Gervain et al., 2011; Ferrari and Quaresima, 2012). A single emitter–detector pair was positioned according to the international 10/10 electrode placement system so that the midpoint was centered over either C1 or C2 (whichever was contralateral to the stimulation site; Fig. 1*A*) to provide access to the representation area of the heel in the primary somatosensory cortex (Fig. 1*B*). The emitter was always placed toward the front of the head, and the emitter–detector separation was kept constant at 4 cm using a commercial probe holder (Hamamatsu Photonics K.K.), which was held in place using an elastic net (Surgifix, FRA Production SpA) and a baby hat. Figure 1*B* shows that with this separation, an emitter–detector pair centered over C1 in 40-week-old infants is sensitive to large areas of SI, including the heel area. Since NIRS is mainly sensitive to the microvasculature (Schroeter et al., 2006; Ferrari and Quaresima, 2012), the presence of the superior sagittal sinus in the midline region is unlikely to influence the evoked hemodynamic responses. The sensitivity maps in Figure 1*B* were produced using the four-dimensional neonatal head model described in the study by Brigadoi et al. (2014).

#### EEG recording

Recording electrodes (disposable Ag/AgCl cup electrodes) were positioned according to a modified international 10/20 electrode placement system at Fp1, Fp2, Fz, F3, F4, Cz (vertex), C3, C4, CPz, CP3, CP4, T3, T4, T5, T6, O1, O2, and POz (Fig. 1*A*). Although it was not always possible to apply the full set of electrodes, in the majority of infants at least 12 electrodes were used, and the Cz electrode was used in all recordings. Reference and ground electrodes were placed at FCz and on the forehead, respectively. Electrode–skin impedance was kept to a minimum by rubbing the skin with an EEG prepping gel (NuPrep Gel, Weaver and Company) and contact with the electrodes was optimized by applying conductive EEG paste (Ten20, Weaver and Company; or Elefix, Nihon Kohden). Electrodes were held in place using an elastic net (Surgifix, FRA Production SpA), and electrode leads were tied together to minimize electrical interference. EEG activity, from DC to 70 Hz, was recorded using the Neuroscan SynAmps2 EEG/EP Recording System (Compumedics Neuroscan). A 50 Hz notch filter was used, and signals were digitized with a sampling rate of 2 kHz and a resolution of 24 bits.

All EEG recordings were reported as normal by a clinical physiologist (A.L.) with respect to symmetry, synchronicity, absence of epileptiform activity, and background rhythms appropriate for age. The infants’ sleep state was also classified as either “awake” or “asleep” using electrophysiological and behavioral data. Eighteen of 21 infants who received a heel lance were classified as asleep prior to the heel lance; 1 infant was awake and 2 infants could not be classified.

### NIRS analysis

Concentration changes in oxyhemoglobin (Δ[HbO_2_]), deoxyhemoglobin (Δ[HHb]), and total hemoglobin (HbT; calculated as Δ[HbT] = Δ[HbO_2_] + Δ[HHb]) were analyzed using custom-written MATLAB (MathWorks) scripts. Traces were band-pass filtered between 0.05 and 1 Hz (using a first-order Butterworth filter) and were segmented into 40 s epochs starting from 20 s prior to each stimulus. Each epoch was then baseline corrected using the prestimulus interval. HbO_2_, HHb, and HbT traces were assessed for movement artifacts; epochs containing large-amplitude movement artifacts (defined as Δ[HbT] > 25 µm) were rejected, and epochs containing brief (<3 s duration), low-amplitude (<15 µm) artifactual spikes were interpolated (at the point of inflection, using piecewise cubic spline interpolation).

Three lance and two control epochs were rejected for technical reasons (e.g., poor quality data due to poor light shielding; failed time locking of the stimulus to the NIRS recording), and a further three lance epochs and one control epoch were rejected due to large-amplitude movement artifacts. Therefore, 15 lance and 18 control epochs were included in the final NIRS sample.

For touch, epochs were rejected if they contained a movement artifact (>5 µm; 18 trials). Five epochs were also rejected as outliers. Thus, a total of 131 touch epochs from 16 participants were considered in the final NIRS sample (Table 2).

**Table 2.** Number of infants included in the analysis

	**Lance**	**Control**	**Touch**
Total sample included	17	20	16 (145)
NIRS accepted	15	18	16 (131)
EEG accepted	16	16	11 (106)

Data refer to the number of infants; parentheses indicate the number of touches across all infants.

To determine whether we could record a hemodynamic response to noxious, control, and tactile stimuli at a group level, we computed the group average Δ[HbO_2_], Δ[HHb], and Δ[HbT] for each stimulus type, and performed *z*-tests at each time point from 0 to 20 s poststimulation. In order to account for multiple testing, only significant segments of at least 1 s duration were considered to be meaningful. This allowed us to identify when the hemodynamic response exceeded random baseline noise. For touch, epochs were first averaged within individual participants, and then across participants to give a grand average. Hemodynamic responses to each stimulus were characterized from the group averages in terms of peak changes and latencies.

To compare the hemodynamic response to lance with the response to control or touch stimulation, independent-samples *t* tests were then performed at each time point from 0 to 20s poststimulation. Corrections for multiple comparisons were performed as described above.

In adults, a typical hemodynamic response consists of an increase in [HbO_2_] and a concomitant, lower-amplitude decrease in [HHb], reflecting an increase in cerebral blood flow to the activated region, and the resulting oversupply of HbO_2_ and displacement of HHb from the veins. However, in infants the direction of the Δ[HHb] is not consistent across studies, with some studies reporting decreases and others increases in [HHb], or inconsistent results (for review, see Lloyd-Fox et al., 2010a). To determine the dominant peak of the HHb and HbO_2_ responses for each trial in our data, we looked for changes that exceeded a threshold of 2 SDs for at least 1 s from the mean baseline of the given trial between 1 and 6.5 s after stimulation.

For all tests, the threshold for significance was set at α = 0.05.

### Event-related potential analysis

Five lance and five control trials were removed from the analysis for technical reasons (e.g., because the EEG was not performed or was of poor quality; or due to failed time locking of the stimulus to the EEG recording). Therefore, 16 lance and 16 control trials were included in the final EEG sample.

For touch, five test occasions and a further three trials from three other test occasions were excluded for technical reasons (e.g., because the EEG was not performed or was of poor quality). Thus, a total of 106 touch trials from 11 participants were included in the final EEG sample (Table 2).

EEG traces were analyzed using EEGLAB (Delorme and Makeig, 2004) and custom-written MATLAB (MathWorks) scripts. Traces were band-pass filtered between 1 and 30 Hz (using a second-order bidirectional Butterworth filter), segmented into 1.7 s epochs starting from 0.6 s before the stimulus, and baseline corrected using the prestimulus interval. Channels containing a movement artifact (defined as activity exceeding ±100 µV) or high-frequency muscle activity were removed.

The analysis focused on Cz. In order to correct for intertrial and intersubject latency jitter (Woody, 1967; Bromm and Scharein, 1982), traces were aligned by Woody filtering within time windows centered on the N2P2 and N3P3 waveforms, as follows: (1) 50–400 ms after stimulation for lance epochs or 50–300 ms for control and touch epochs; and (2) 350–600 ms after stimulation. The maximum allowed jitter correction was ±50 ms for lance and touch epochs, and ±75 ms for control epochs. This approach resulted in two aligned group averages per stimulus type. For touch trials, traces were first aligned and averaged within participants, and the resulting traces were then aligned and averaged across individuals to give the grand average trace.

To determine whether the waveforms exceeded random baseline noise, *z*-tests were performed at each time point within the alignment windows (50–300 and 350–600 ms). We used the false discovery rate (Benjamini and Hochberg, 1995) to correct for multiple comparisons and assumed 30 independent tests/s, because data were low-pass filtered at 30 Hz.

The amplitude and latency of the negative (N) and positive (P) peaks of the two waveforms were obtained from the aligned group averages. Scalp topography maps were also created from the aligned group averages in order to display the scalp distribution of the N and P peaks of each waveform. For each peak, the average amplitude at Cz and at each of the other channels at the time of the given peak was plotted as a heat map. Channels that were excluded due to contamination by artifacts or were not recorded were interpolated.

The peak-to-peak amplitudes of the N and P peaks of the nociceptive-specific waveform in the lance trials were obtained by comparing each individual trace with the average and selecting the peaks that most resembled the average trace N and P peaks in terms of latency and morphology.

### Within-infant comparison of NIRS and EEG lance responses

After establishing the presence of hemodynamic and electrophysiological responses at the group level, we investigated the relationship between the two measures at the single-trial level. Specifically, we explored whether NIRS and EEG responses co-occurred within the same test occasion. To do so, we checked for the presence of an HbO_2_ response and of the nociceptive-specific EEG waveform in each lance trial, and then compared the two. For control trials, we assessed the presence of the first (non-nociceptive-specific) EEG waveform.

#### NIRS goodness-of-fit analysis

**Figure 2.**
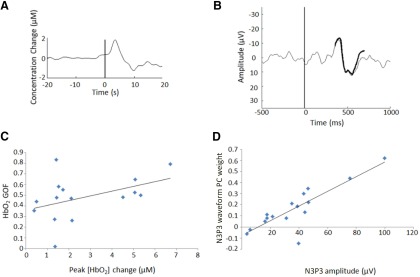
HbO_2_ GOF values and EEG N3P3 waveform PC weights correlate with peak Δ[HbO_2_] and N3P3 amplitudes, respectively, in term infants having a noxious heel lance. ***A***, HRF used for classifying trials according to the presence of an HbO_2_ response. ***B***, The PC used for classifying trials according to the presence of the nociceptive-specific N3P3 waveform is shown in bold, overlaid onto the average EEG response for clarity. ***C***, NIRS HbO_2_ GOF values are plotted against peak positive [HbO_2_] changes, indicating a positive correlation that is almost significant (Spearman’s ρ = 0.51, *p* = 0.052, *n* = 15). ***D***, EEG N3P3 waveform PC weights are plotted against the N3P3 amplitudes, indicating a significant positive correlation (Spearman’s ρ = 0.81, *p* = 0.0002, *n* = 16).

To establish whether an HbO_2_ response was present in a given trial, we compared each epoch to a hemodynamic response function (HRF) template. This was modeled as the average Δ[HbO_2_] of all lance epochs, as in the NIRS analysis section, and smoothed with an automatic one-dimensional wavelet denoising filter in MATLAB (threshold selection was based on Stein’s Unbiased Risk Estimate, and additional parameters included soft thresholding and no rescaling). This yielded a lance HRF template with latency to a positive peak of 3.4 s, a positive peak amplitude of 1.8 µm, a latency to an undershoot peak of 9.8 s, and a ratio of undershoot amplitude to positive peak amplitude of 0.72 (Fig. 2*A*). The same procedure applied to the control trials produced a control HRF template with a latency to positive peak of 2.2 s, a positive peak amplitude of 0.3 µm, a latency to an undershoot peak of 12.6 s, and a ratio of undershoot amplitude to positive peak amplitude of 1.07. Each individual [HbO_2_] trace was correlated with the HRF to determine the GOF between the observed and expected Δ[HbO_2_]. NIRS epochs were classified as “response present” if they exceeded a GOF threshold of 0.45, and “response absent” otherwise.

#### EEG principal component analysis

We then assessed the presence of the tactile and the nociceptive specific ERPs in the same lance and control trials. This was accomplished using PCA (Slater et al., 2010a). The tactile- and nociceptive-specific components were identified by conducting PCA in the following two time intervals: (1) 50–300 ms poststimulation (lance and control trials separately); and (2) 350–700 ms poststimulation (lance trials only). The tactile- or nociceptive-specific waveform was considered to be present in a given EEG epoch if the weight associated with the corresponding component exceeded a threshold of 0.1, and was considered absent otherwise.

**Figure 3.**
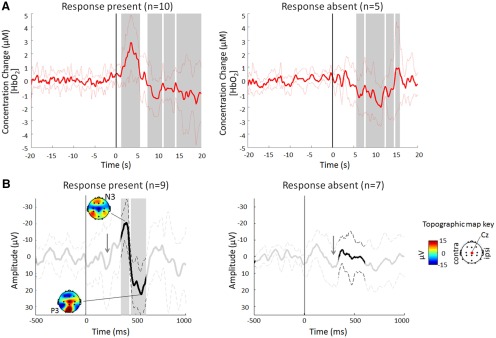
***A***, ***B***, Nociceptive-specific responses can be identified in the average of NIRS (***A***) and EEG (***B***) trials classified as response present, but not in the average of those trials classified as response absent. ***A***, Average (±SD) Δ[HbO_2_] following noxious heel lance (*t* = 0 s) at the contralateral primary somatosensory cortex in 10 response-present (left) and 5 response-absent (right) trials. ***B***, Average (±SD) ERP at Cz following noxious heel lance (*t* = 0 ms) in nine response-present (left) and 7 response-absent (right) trials, with topography maps at the N and P peaks. Gray arrows indicate the location of the first waveform. Time points that are significantly different from baseline are highlighted in gray.

Using these criteria, responses were classified as present in 10 of 15 lance NIRS trials (GOF >0.45), and in 9 of 16 lance EEG trials [principal component (PC) weight >0.1]. The accuracy of this classification is confirmed by the fact that a significant response is present in the group averages of the trials where the response was “present,” but not in that of the trials where the response was “absent” (Fig. 3). Finally, even though our method is preferable to using peak amplitudes, because it takes into account the overall signal rather than a single data point that can be affected by noise, the GOF values^a^ and PC weights^b^ correlated well with the more traditional peak measures (Fig. 2*C*,*D*).

## Results

### Distinct hemodynamic responses to noxious heel lance and innocuous heel touch in newborn infants: group analysis

**Figure 4.**
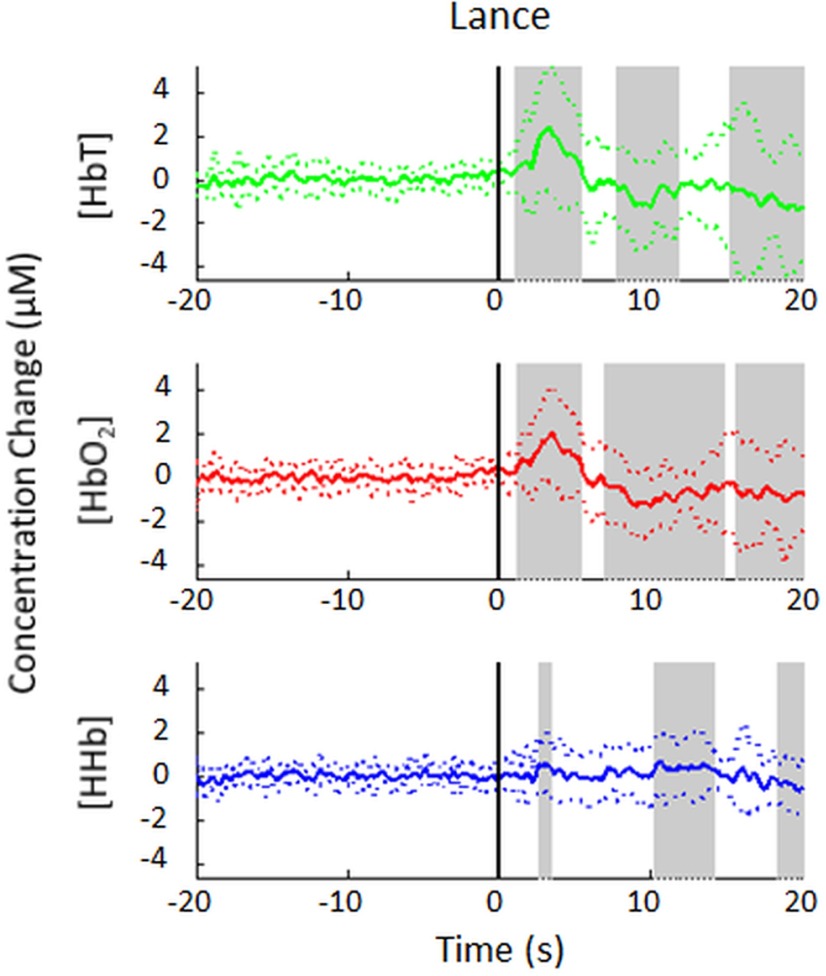
Average (±SD) hemodynamic response to a noxious heel lance (*t* = 0 s) at the contralateral primary somatosensory cortex in 15 term infants. [HbT], [HbO_2_], and [HHb] changes are plotted separately (in green, red, and blue, respectively), and time points that are significantly different from baseline are highlighted in gray.

We first analyzed and compared the hemodynamic response to lance and to innocuous touch of the heel. NIRS analysis was performed in 15 infants undergoing a clinically required heel lance (*n* = 9 right heel lances). Consistent with previous studies (Bartocci et al., 2006; Slater et al., 2006), a significant increase of 2.3 ±2.9 µm in [HbT] was recorded over the contralateral primary somatosensory cortex. The response had a maximum peak at 3.2 s, and was followed by a significant undershoot at 10 s and a later decrease from 15.2 s (Fig. 4; Table 3).

**Table 3.** Peak amplitude and latency of the grand average hemodynamic response to lance, control, and touch

	**Early peak**			**Undershoot/overshoot**		
	**[HbT]**	**[HbO_2_]**	**[HHb]**	**[HbT]**	**[HbO_2_]**	**[HHb]**
Lance						
Amplitude, µm	2.3 ± 2.9	2.0 ± 2.2	0.5 ± 1.5	−1.3 ± 1.9	−1.3 ± 1.5	0.7 ± 1.4
Latency, s	3.2	3.4	3.2	10	9	10.6
Control						
Amplitude, µm	0.6 ± 1.1	0.4 ± 0.6	NS	−0.5 ± 0.8	−0.5 ± 0.6	NS
Latency, s	2.8	2.2	NS	12.4	13.4	NS
Touch						
Amplitude, µm	0.2 ± 0.3	0.3 ± 0.2	NS	NS	NS	−0.1 ± 0.2
Latency, s	2.8	4.0	NS	NS	NS	9.8

Amplitude is shown as the mean ± SD. Early peak, initial response occurring between 1.0 and 6.5 s; undershoot/overshoot, next identifiable peak occurring after 6.5 s; NS, not significant.

The Δ[HbT] largely reflects the Δ[HbO_2_], which had the same statistically significant response pattern (Fig. 4; Table 3). Since the HHb response was small and variable (with increases in the early HHb response in 6 of 15 trials; decreases in 4 of 15 trials; and no change in 5 of 15 trials), as is well known from other infant studies (Lloyd-Fox et al., 2010a), [HbO_2_] changes (10 of 15 increases; 1 of 15 decreases; 4 of 15 no change) were used for subsequent analysis.

**Figure 5.**
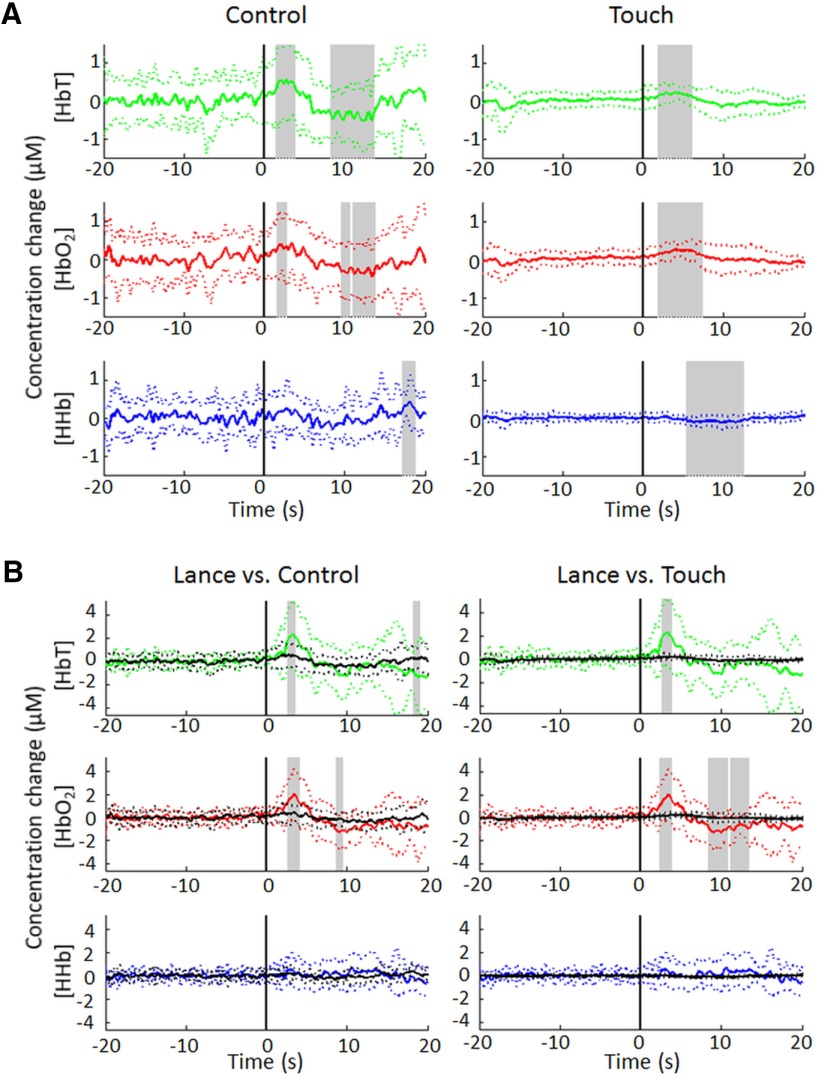
Noxious stimulation elicits a more pronounced hemodynamic response than innocuous control or touch stimulation. ***A***, Average (±SD) hemodynamic response to non-noxious control (left, *n* = 18 infants) and touch (right, *n* = 131 touches from 16 infants) at the contralateral primary somatosensory cortex. ***B***, Results of an independent-samples *t* test comparing lance (black traces; *n* = 15 infants) and control (left) or touch (right). Average (±SD) [HbT], [HbO_2_], and [HHb] changes are plotted separately (in green, red, and blue, respectively), and statistically significant differences from baseline (***A***) or between stimuli (***B***) are highlighted in gray.

NIRS analysis was also performed on infants undergoing non-noxious control stimulus (18 infants, *n* = 9 right side) or repeated touch of the heel (*n* = 16 infants, 131 trials, mean = 7 trials per infant, *n* = 10 right side). As with lance stimulation, there was a significant early increase in [HbO_2_] following both control and touch stimuli, although this increase was markedly lower in amplitude compared with lance stimulation (2.0 ± 2.2 µm following lance vs. 0.4 ± 0.6 and 0.3 ± 0.2 µm following control and touch stimulation, respectively; Fig. 5*A*; Table 3). For the control stimulus, this peak was followed by a significant undershoot peaking at 13.4 s. There was no significant early Δ[HHb] following either control or touch, although there was a late decrease in [HHb], peaking at 9.8 s following touch stimulation.

The hemodynamic response to noxious heel lance was significantly larger than the response to innocuous mechanical skin stimulation. Independent-samples *t* tests confirmed that both the early increase in [HbO_2_] and the ensuing undershoot were significantly larger following lance than either control or touch. No significant differences were observed in HHb responses (Fig. 5*B*).

### Distinct EEG responses to noxious and innocuous heel stimulation in newborn infants: group analysis

**Figure 6.**
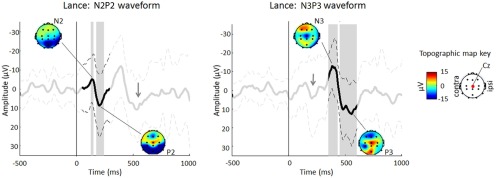
Average (±SD) EEG response at Cz following noxious heel lance (*t* = 0 ms) in 16 term infants when aligned to the first waveform (between 50 and 400 ms; left) and to the second waveform (between 350 and 600 ms; right), with topography maps at the N and P peaks. Time points between 50 and 300 ms (left) and between 350 and 600 ms (right) that are significantly different from baseline are highlighted in gray. Note that the group average responses are from the same group of infants but look different because the individual trials have been aligned differently. Gray arrows indicate the location of the second waveform when traces are aligned to waveform 1 (left) and of the first waveform when traces are aligned to waveform 2 (right).

We next analyzed and compared the EEG responses to lance and to innocuous touch of the heel. Sixteen infants receiving a heel lance were included in the EEG analysis. Heel lance evoked a clear EEG response consisting of a late N2P2 complex followed by an N3P3 complex. Both waveforms were significantly different from baseline (Fig. 6). Following alignment, the mean latencies of the N and P peaks of the first waveform were 139 and 202 ms, and the amplitudes were −5.0 ± 12.2 and 8.7 ± 16.6 µV, respectively. The mean N3 and P3 peaks were 385 and 554 ms in latency, and −12.8 ± 12.1 and 12.7 ± 17.1 µV in amplitude, respectively (Table 4). Scalp topography maps showed that the P2, N3, and P3 peaks were maximal at the vertex. The N peak of the first waveform was instead maximal at POz.

**Table 4.** Peak latency and amplitude of the mean lance, control, and touch ERPs

	**Lance**	**Control**	**Touch**
N2			
Amplitude, µV	−5.0 ± 12.2	−5.1 ± 15.5	−9.1 ± 10.1
Latency, ms	139	93	147
P2			
Amplitude, µV	8.7 ± 16.6	20.1 ± 20.1	9.5 ± 8.4
Latency, ms	202	189	248
N3			
Amplitude, µV	−12.8 ± 12.1		
Latency, ms	385		
P3			
Amplitude, µV	12.7 ± 17.1		
Latency, ms	554		

Data are shown as the mean ± SD.

**Figure 7.**
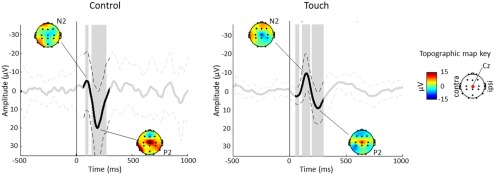
Average (±SD) EEG response at Cz following innocuous control (left; *n* = 16 term infants) and touch (right; *n* = 11 term infants having 106 touch trials) stimulation when aligned to the N2P2 waveform (between 50 and 300 ms), with topography maps at the N and P peaks. Time points between 50 and 300 ms that are significantly different from baseline are highlighted in gray.

The EEG traces of 16 infants having a control stimulus and 11 infants having a total of 106 touches were also analyzed. Both control and touch stimuli elicited a distinct late N2P2 complex. For both stimuli, the N and P peaks were significantly different from baseline (Fig. 7). For the control stimulus, the mean latencies of the N and P peaks following alignment were 93 and 189 ms, respectively, and the amplitudes were −5.1 ± 15.5 and 20.1 ± 20.1 µV. For the touch stimulus, the mean latencies and amplitudes of the N and P peaks were 147 and 248 ms and −9.1 ± 10.1 and 9.5 ± 8.4 µV, respectively (Fig. 7; Table 4). Scalp topography maps showed that the N and P peaks were maximal at the vertex for both the control and touch averages. The late N3P3 EEG response to noxious heel lance was not observed in response to innocuous mechanical skin stimulation.

### Simultaneous NIRS and EEG recordings in response to cutaneous stimulation in newborn infants: individual infant analysis

**Figure 8.**
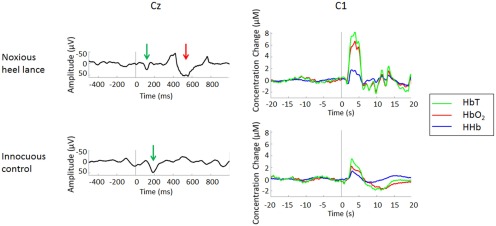
Combined EEG and NIRS recordings can be successfully performed in neonates in response to cutaneous stimulation. Simultaneous EEG (left) and NIRS (right) recordings in a single term infant following noxious heel lance (top) and innocuous control stimulation (bottom; stimulus at *t* = 0 s), showing artifact-free EEG traces at Cz and NIRS traces at C1. Arrows indicate the presence of a nociceptive-specific EEG waveform (red) following heel lance and an earlier EEG waveform (green) following both stimuli. Clear increases in [HbT], [HbO_2_], and [HHb] follow both noxious and innocuous stimulation (right).

The results above show that both NIRS and EEG can be used to record quantifiable and distinct innocuous and noxious evoked activity at a group level in the newborn cortex. We next asked whether such distinct hemodynamic and electrophysiological responses to noxious and innocuous stimulation co-occur in individual trials. In all 18 infants who received a noxious heel lance and a matching control non-noxious stimulus (lancet rotated 90°), artifact-free NIRS and EEG recordings were successfully obtained (Fig. 8), but four lance trials were rejected for technical failure or movement artifact during the noxious procedure.

Eleven of 14 lance trials contained a cortical nociceptive response (3 HbO_2_ only; 2 ERP only; 6 both), while in 3 trials no response was detected in either HbO_2_ or ERP recordings, according to the stringent criteria described in Materials and Methods. Thus, although cortical NIRS and EEG responses to noxious heel lance can be recorded simultaneously, and in the majority of test occasions the two methods are consistent [present or absent together, 9 of 14 trials (64%); Table 5], they do not always co-occur.

**Table 5.** Classification of NIRS HbO_2_ and EEG waveform 2 responses

		**NIRS HbO_2_**		
		**Present**	**Absent**	
EEG	Present	**6**	2	**8**
N3P3	Absent	3	**3**	6
		**9**	5	14

**Table 6.** Statistical table

**Location**	**Data structure**	**Type of test**	**Confidence interval**
a (Fig. 2C)	HbO_2_ GOF values but not HbO_2_ peak values are normally distributed	Spearman’s ρ	−0.03 to 0.86
b (Fig. 2D)	N3P3 PC weights but not N3P3 amplitudes are normally distributed	Spearman’s ρ	0.39–0.98
c	HbO_2_ GOF values and N3P3 PC weights are both normally distributed	Pearson’s *r*	0.15–0.96
d	HbO_2_ GOF values but not N2P2 PC weights are normally distributed	Spearman’s ρ	−0.22 to 0.89

The noxious evoked HbO_2_ response showed less co-occurrence with the N2P2 component of the noxious evoked EEG response [present or absent together, 5 of 14 trials (35.7%)], suggesting that the hemodynamic response was better related to the nociceptive-specific component of the lance EEG response.

Three control trials were rejected for technical failure or movement artifact during stimulation. Eleven of 15 test occasions contained a cortical touch response (3 HbO_2_ only; 5 ERP only; 3 both), while 4 contained neither an HbO_2_ nor an ERP response. Thus, although cortical NIRS and EEG responses to innocuous touch can be recorded simultaneously, the two methods are not consistent [present or absent together, 7 of 15 trials (47%)], and they do not always co-occur.

### Cortical NIRS and EEG responses to noxious heel lance and to innocuous control are correlated

**Figure 9.**
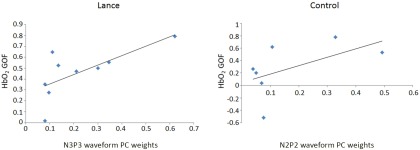
Hemodynamic and EEG responses to innocuous control and to noxious heel lance are related. Scatterplots show a strong positive correlation between hemodynamic and EEG responses for the lance (left, *n* = 9) and control (right, *n* = 7) trials that were classified in the same way by the two cortical measures.

Despite the fact that they did not always co-occur, we predicted that the HbO_2_ GOF values and the PC weights of the N3P3 nociceptive-specific waveform would be correlated when they did co-occur. In the nine trials where the incidence of the two cortical measures agreed, a significant positive correlation between HbO_2_ GOF values and the PC weights of the nociceptive-specific N3P3 waveform was found^c^ (Pearson’s *r* = 0.69, *p* = 0.04; Fig. 9). Similarly, there was a positive correlation between HbO_2_ GOF values and the PC weights of the N2P2 waveform of the seven control trials where the incidence of the two cortical measures agreed, although this was not significant^d^ (Spearman’s ρ = 0.54, *p* = 0.215; Table 6).

## Discussion

Here, we have recorded, for the first time, simultaneous hemodynamic and neurophysiological responses to innocuous control and tactile stimuli and to noxious heel lance in newborn human infants. We fully characterized hemodynamic responses in terms of both [HbO_2_] and [HHb] changes, and developed a method to identify the presence of a clear hemodynamic response to cutaneous stimuli on a single-trial basis. The magnitude of the hemodynamic responses reported here is in the same range as those reported elsewhere in infants, in response to somatosensory (Slater et al., 2006), visual (Meek et al., 1998), olfactory (Bartocci et al., 2000), and other stimulation (Lloyd-Fox et al., 2009, 2010b). As predicted, we observed significant hemodynamic responses to both noxious and innocuous stimuli that also elicited clear ERPs in a group of healthy term infants. Importantly, we also found significantly greater hemodynamic activation in the contralateral SI following lance compared with control or tactile stimulation, indicating the selectivity of hemodynamic responses. However, our hypothesis that neural and hemodynamic responses would always co-occur in individual trials was not supported, although in the majority of noxious lance trials they did coincide and were positively correlated.

This is the first clearly documented quantitative analysis of both innocuous and noxious evoked hemodynamic activity in the infant human brain. Previous studies reported no HbT response to von Frey hair stimulation of the foot (Slater et al., 2006), which appears inconsistent with the present results. Our finding here of a significant increase in both [HbO_2_] and [HbT] following a similarly brief innocuous mechanical stimulus suggests that the ability to detect a hemodynamic response does not depend upon stimulus duration or which hemoglobin signal is measured. Instead, this inconsistency is likely due to the smaller hemodynamic response and lower signal-to-noise ratio of the tactile response relative to the noxious response, which was the focus of the earlier study. A previous report of specific somatosensory hemodynamic responses in the infant cortex (Bartocci et al. 2006) used longer-duration skin wiping as a stimulus, but the lack of detailed methodology and analysis makes the results of that study hard to compare with the current results.

### Distinct tactile and nociceptive cortical responses at the group level

Our results show initial increases in [HbO_2_] over the contralateral SI following lance, control, and tactile stimuli (2.0, 0.4, and 0.3 µm, respectively). Furthermore, hemodynamic responses were modulated such that it was possible to distinguish between noxious lance and the innocuous control and tactile stimuli based on the magnitude of the [HbO_2_] changes. The mean evoked noxious response was almost 10 times larger in amplitude than the response to innocuous stimulation. This is a considerably greater difference than that reported in a previous NIRS study using innocuous and noxious electrical stimulation in healthy adults (Yücel et al., 2015). Interestingly, other adult studies using fMRI and diffuse optical tomography (a technique that uses NIRS to enable volumetric monitoring of [HbO_2_] and [HHb] changes), show that noxious and innocuous stimuli can be differentiated only with respect to the shape and bilaterality, rather than the magnitude, of responses (Chen et al., 2002; Becerra et al., 2008, 2009).

This study also confirms the presence of a distinct late nociceptive N3P3 waveform in the infant brain following noxious stimulation that is clearly separable from an earlier N2P2 wave associated with innocuous mechanical stimulation (Slater et al., 2010a; Fabrizi et al., 2011; Verriotis et al., 2015). Thus, we show here that both techniques are able to distinguish between painful and nonpainful cortical activation (NIRS in terms of peak amplitudes and EEG in separable waveforms).

### Interpreting the hemodynamic response

We did not find significant differences in [HHb] changes between stimuli; this could be due to the well known variable nature of [HHb] changes in young infants (Lloyd-Fox et al., 2010a). In adults, increased oxygen consumption leads to regional overperfusion, resulting in a net increase in [HbO_2_] and a decrease in [HHb]. The variability in the Δ[HHb] seen in infants suggests that increased oxygen consumption does not always lead to regional overperfusion, perhaps due to immature vascular regulation or to greater metabolic demands of neurotransmission in unmyelinated white matter (Meek, 2002; Gervain et al., 2011; Kozberg et al., 2013).

In addition to the early changes in [HbO_2_] and [HHb], we observed a significant [HbO_2_] decrease from 7 s onward until the end of the 20 s trace following lance, and a similar but shorter lasting decrease following control stimulation (Figs. 4, 5; Table 3). This is consistent with the HbO_2_ “undershoot” (and concomitant HHb “overshoot”) that has been observed in other NIRS studies (Obrig et al., 2000; Steinbrink et al., 2006; Boden et al., 2007; Gervain et al., 2011) as well as with the BOLD undershoot reported in fMRI studies, which has been observed for up to 60 s (Schroeter et al., 2006; Steinbrink et al., 2006; Chen and Pike, 2009; Arichi et al., 2012). There was also a significant average [HHb] increase between 10 and 14 s following lance, but this is difficult to interpret as either an overshoot or undershoot because the direction of the early HHb response was variable. The physiological origin of an [HbO_2_] undershoot and an [HHb] overshoot has been explored in several studies, and is controversial due to the existence of evidence supporting different mechanisms (Schroeter et al., 2006; Steinbrink et al., 2006; Chen and Pike, 2009). The variable HHb response in our study, in addition to the developmental maturation effects of neurovascular coupling and energy use on the hemodynamic response in young infants (Harris et al., 2011; Kozberg et al., 2013), makes it hard to associate our data with any of these mechanisms.

The latencies of the peak hemodynamic responses to lance, control, and tactile stimuli are relatively short (3.4, 2.2, and 4.0 s, respectively, for [HbO_2_] changes), as reported elsewhere (Slater et al., 2006). In adult fMRI studies, the canonical HRF in response to a stimulus is typically modeled with a peak latency of ∼5–6 s (Henson, 2004), although peak latencies of 4–5 s have been reported (Steffener et al., 2010); whereas in infants hemodynamic responses tend to peak later (Meek, 2002; Arichi et al., 2012). The shorter peak latencies reported here may be due to differences in stimulus durations (∼5–300 ms vs 3.2–30 s; Meek et al., 1998; Taga et al., 2003; Bartocci et al., 2006; Wartenburger et al., 2007; Lloyd-Fox et al., 2009), as well as age, type of stimulus, technical parameters, and analysis methods (e.g., filtering methods). Another explanation is that immature vascular regulation, and therefore neurovascular coupling, in the neonates may result in reduced hyperemia relative to adults (Meek, 2002; Gervain et al., 2011; Harris et al., 2011; Kozberg et al., 2013), leading to a shorter lasting increase in [HbO_2_] and, therefore, a shorter peak latency.

It should be noted that, in adults, noxious stimuli can trigger a generalized sympathetic skin response in addition to a cortical response (Yücel et al., 2015). While an effect of this autonomic response on the hemodynamic changes observed in the present study cannot be conclusively ruled out without monitoring superficial skin activity, the present results are likely to reflect cortical activity, for four reasons. First, the results reported by Yücel et al. (2015) suggest that the superficial response would be in the opposite direction to the [HbO_2_] changes reported here, thus masking the cortical response (Yücel et al., 2015). Second, consistent with other studies, hemodynamic responses to noxious heel lance were in the same direction as the responses to tactile stimuli, which are less likely to trigger an autonomic skin response. Third, as neonates have a much thinner scalp and skull than adults, the relative contribution of superficial skin activity to the measured signal would be much smaller in neonates; indeed, the optimal emitter–detector separation required to sample scalp activity without cortical activity is 2.15 mm in term neonates versus 8.4 mm in adults (Brigadoi and Cooper, 2015). Finally, NIRS responses to heel lance are consistent with those reported in a previous study (Slater et al., 2006) in which simultaneously monitored ipsilateral responses were variable and often in the opposite direction, indicating that heel lance elicited localized rather than global hemodynamic changes.

### Interpretation of simultaneous NIRS-EEG recordings

Several combined NIRS-EEG recordings have been recently undertaken in infants (Roche-Labarbe et al., 2007, 2008; Telkemeyer et al., 2009; Biallas et al., 2012), and there is great interest in developing probes for this purpose (Cooper et al., 2009; Wallois et al., 2012). Here we report the first combined EEG and NIRS analysis of infant somatosensory cortical activity. As predicted, we found that somatosensory stimuli elicit both neural and hemodynamic responses in a given group of infants. However, single-trial analysis showed that neural and hemodynamic responses do not co-occur in all trials. This is true following both innocuous control and noxious lance stimuli, although neural and hemodynamic responses co-occurred in a greater proportion of trials following lance. For both stimuli, an ERP was observed in the absence of a hemodynamic response over the contralateral SI in some trials, and a hemodynamic response was detected in the absence of an ERP in other trials.

In adults, a network of brain regions, including the SI, is implicated in the generation of the noxious laser-evoked potential (Garcia-Larrea et al., 2003; Valentini et al., 2012; Hu et al., 2014). The co-occurrence of neural and hemodynamic responses in many of the trials suggests that the contralateral SI also contributes to the generation of the nociceptive-specific ERP in the maturing newborn brain, but is not reliably activated every time at this age. Similarly, the presence of a hemodynamic response in the absence of an ERP could be interpreted as intact involvement of the SI but insufficient involvement of the other generators contributing to the ERP. Alternatively, the presence of a clear HbO_2_ response in the absence of an ERP could be explained by the sensitivity of the two techniques to slightly different populations of cells. NIRS can detect hemodynamic activity arising from highly metabolically active basket and cortical stellate cells, which may well be invisible in scalp EEG recordings (Wallois et al., 2012). Moreover, NIRS can detect hemodynamic activity arising from both synchronous and asynchronous neural activity, whereas EEG requires several square centimeters of synchronously active brain tissue in order to detect a response (Wallois et al., 2012). Thus, a poorly organized neural response, as might be likely in the immature cortex, could generate metabolic demands that are sufficient to cause a detectable hemodynamic response, but not an ERP.

Similarly, the presence of an ERP in the absence of a hemodynamic response could be related to immature neurovascular coupling in this age group. Both the coupling between blood flow and neuronal activation, and the regulation of blood flow itself, might be less efficient at this age, such that the effects of increased oxygen consumption and increased blood flow cancel each other out and no hemodynamic response is observed (Harris et al., 2011). Therefore, even if the SI is one of the generators of the ERP, a hemodynamic response might not always be detectable in the immature brain. A lack of recruitment of pial arteries in early development (resulting in little or no initial hyperemia), but later vasoconstriction (resulting in an apparent inverted hemodynamic pattern), is one potential mechanism of the immature neurovascular coupling (Kozberg et al., 2013). Alternative explanations include masking of the hemodynamic response in some cases due either to autonomic sympathetic activity, which is known to follow noxious stimulation (Yücel et al., 2015), or to the sensitivity of the optodes to a large area of cortex, parts of which could be deactivated.

### Interpreting trials with no cortical response

It is clear from this study that the lack of a detectable NIRS or a detectable EEG response alone does not imply that noxious input was not processed in the cortex of these infants. Nonetheless, the proportion of infants having no detectable NIRS response to noxious heel lance (5 of 14) is larger than that reported elsewhere (1 of 18; Slater et al., 2006). This discrepancy is probably due to the more stringent classification method used here, and to the large number of babies that were asleep, leading to a reduced hemodynamic response (Slater et al., 2006). The proportion of infants with no EEG response is expected; a clear lance ERP is not always detected in neonates, as the ERP begins to appear more reliably just before the time of normal birth, and its incidence continues to increase with age until at least 45 weeks GA (Fabrizi et al., 2011) and in amplitude until at least 1 year of age (Verriotis et al., 2015).

However, in a small number of infants there was neither an EEG nor a NIRS response to somatosensory stimulation, be it noxious or innocuous, despite clear evidence of high-quality artifact-free recording and of behavioral reactivity (data not shown). It is likely that a lack of either response reflects genuine intertrial and interindividual variability in pain reactivity in infants. The reproducibility of within-subject responses in key somatosensory regions to tactile and painful stimuli is known to be not entirely stable in healthy adults (Taylor and Davis, 2009) and is likely to be less so in infants. But there is also increasing evidence of interindividual differences in pain perception influenced by both genetic and epigenetic factors. A recent study has highlighted the significant variability of infant pain behavior between groups of infants who were 2–12 months of age (Pillai Riddell et al., 2013), which is consistent with the considerable variability in facial expression following heel lance in preterm and term infants on a given study occasion (Slater et al., 2009).

Our ability to record and quantify single-trial hemodynamic and EEG responses increases the reliability of either method alone and will allow us to interrogate these responses in the maturing infant brain and to better interpret developmental changes in central pain processing.

## Synthesis

The decision was a result of the Reviewing Editor Karen Davis and the peer reviewers coming together and discussing their recommendations until a consensus was reached. A fact-based synthesis statement explaining their decision and outlining what is needed to prepare a revision is listed below. The following reviewers agreed to reveal their identity: Lino Becerra, Markus Ploner

Pain processing in the infant brain is an interesting and clinically relevant topic. The present study investigates brain responses to pain and touch in human infants. The investigators simultaneously recorded electroencephalography (EEG) and near-infrared spectroscopy (NIRS) responses to painful heel lance (for diagnostic purposes) and non-painful stimuli in 30 newborn infants. The results show known patterns of EEG and NIRS responses to both stimuli. Single trial analysis further revealed that brain responses cannot be recorded in all trials and that the detection of EEG responses does not necessarily co-occur with the detection of NIRS responses. The authors concluded that both techniques provide complementary information on somatosensory processing in the infant brain and that the multimodal assessment allows for novel insights in these processes.

This paper is the first to combine both measures and systematically study noxious, innocuous and control evoked stimuli in infants. The study shows that electroencephalography (EEG) and near-infrared spectroscopy (NIRS) provide complementary information on somatosensory processing in the infant brain. Hence, integrated EEG- and NIRS-based assessments promise to further the understanding of pain and touch processing in the infant brain.

The manuscript is well written. The methods are sound and the results are clear and convincing. The interpretation is adequate.

There is a concern with respect to the NIRS data and analysis; as mentioned by the authors, they believe that no contribution will arise from the midline arteries. However, it is well known that systemic changes in blood flow appear in the skin and they will affect the observed signal. The authors do not address this issue in the discussion and should provide a convincing argument that their measurements were not affected by spurious skin signals.

A minor edit is to modify Figure 1b which should have a scale (spatial).

## References

[B1] Arichi T, Fagiolo G, Varela M, Melendez-Calderon A, Allievi A, Merchant N, Tusor N, Counsell SJ, Burdet E, Beckmann CF, Edwards AD (2012) Development of BOLD signal hemodynamic responses in the human brain. Neuroimage 63:663–673. 10.1016/j.neuroimage.2012.06.054 22776460 PMC3459097

[B2] Arridge SR (1995) Photon-measurement density functions. Part I: analytical forms. Appl Opt 34:7395–7409. 21060614 10.1364/AO.34.007395

[B3] Bartocci M, Winberg J, Ruggiero C, Bergqvist LL, Serra G, Lagercrantz H (2000) Activation of olfactory cortex in newborn infants after odor stimulation: a functional near-infrared spectroscopy study. Pediatr Res 48:18–23. 10.1203/00006450-200007000-00006 10879795

[B4] Bartocci M, Bergqvist LL, Lagercrantz H, Anand KJS (2006) Pain activates cortical areas in the preterm newborn brain. Pain 122:109–117. 10.1016/j.pain.2006.01.015 16530965

[B5] Becerra L, Harris W, Joseph D, Huppert T, Boas DA, Borsook D (2008) Diffuse optical tomography of pain and tactile stimulation: activation in cortical sensory and emotional systems. Neuroimage 41:252–259. 10.1016/j.neuroimage.2008.01.047 18394924 PMC2728450

[B6] Becerra L, Harris W, Grant M, George E, Boas D, Borsook D (2009) Diffuse optical tomography activation in the somatosensory cortex: specific activation by painful vs. non-painful thermal stimuli. PLoS One 4:e8016 10.1371/journal.pone.0008016 19956637 PMC2778627

[B7] Benjamini Y, Hochberg Y (1995) Controlling the false discovery rate: a practical and powerful approach to multiple testing. J R Stat Soc Series B Stat Methodol 57:289–300.

[B8] Biallas M, Trajkovic I, Hagmann C, Scholkmann F, Jenny C, Holper L, Beck A, Wolf M (2012) Multimodal recording of brain activity in term newborns during photic stimulation by near-infrared spectroscopy and electroencephalography. J Biomed Opt 17:0860111–0860118. 10.1117/1.JBO.17.8.086011 23224198

[B9] Boden S, Obrig H, Köhncke C, Benav H, Koch SP, Steinbrink J (2007) The oxygenation response to functional stimulation: is there a physiological meaning to the lag between parameters? Neuroimage 36:100–107. 10.1016/j.neuroimage.2007.01.045 17400478

[B10] Brigadoi S, Cooper RJ (2015) How short is short? Optimum source-detector distance for short-separation channels in functional near-infrared spectroscopy. Neurophotonics 2:025005 10.1117/1.NPh.2.2.025005 26158009 PMC4478880

[B11] Brigadoi S, Aljabar P, Kuklisova-Murgasova M, Arridge SR, Cooper RJ (2014) A 4D neonatal head model for diffuse optical imaging of pre-term to term infants. Neuroimage 100:385–394. 10.1016/j.neuroimage.2014.06.028 24954280

[B12] Bromm B, Scharein E (1982) Principal component analysis of pain-related cerebral potentials to mechanical and electrical stimulation in man. Electroencephalogr Clin Neurophysiol 53:94–103. 6173204 10.1016/0013-4694(82)90109-2

[B13] Chen JJ, Pike GB (2009) Origins of the BOLD post-stimulus undershoot. Neuroimage 46:559–568. 10.1016/j.neuroimage.2009.03.015 19303450

[B14] Chen J-I, Ha B, Bushnell MC, Pike B, Duncan GH (2002) Differentiating noxious- and innocuous-related activation of human somatosensory cortices using temporal analysis of fMRI. J Neurophysiol 88:464–474. 12091568 10.1152/jn.2002.88.1.464

[B15] Cooper RJ, Everdell NL, Enfield LC, Gibson AP, Worley A, Hebden JC (2009) Design and evaluation of a probe for simultaneous EEG and near-infrared imaging of cortical activation. Phys Med Biol 54:2093. 10.1088/0031-9155/54/7/016 19287076

[B16] Delorme A, Makeig S (2004) EEGLAB: an open source toolbox for analysis of single-trial EEG dynamics including independent component analysis. J Neurosci Methods 134:9–21. 10.1016/j.jneumeth.2003.10.009 15102499

[B17] Fabrizi L, Slater R, Worley A, Meek J, Boyd S, Olhede S, Fitzgerald M (2011) A shift in sensory processing that enables the developing human brain to discriminate touch from pain. Curr Biol 21:1552–1558. 10.1016/j.cub.2011.08.010 21906948 PMC3191265

[B18] Ferrari M, Quaresima V (2012) A brief review on the history of human functional near-infrared spectroscopy (fNIRS) development and fields of application. Neuroimage 63:921–935. 10.1016/j.neuroimage.2012.03.049 22510258

[B19] Garcia-Larrea L, Frot M, Valeriani M (2003) Brain generators of laser-evoked potentials: from dipoles to functional significance. Neurophysiol Clin Neurophysiol 33:279–292. 14678842 10.1016/j.neucli.2003.10.008

[B20] Gervain J, Mehler J, Werker JF, Nelson CA, Csibra G, Lloyd-Fox S, Shukla M, Aslin RN (2011) Near-infrared spectroscopy: a report from the McDonnell infant methodology consortium. Dev Cogn Neurosci 1:22–46. 10.1016/j.dcn.2010.07.004 22436417 PMC6987576

[B21] Harris JJ, Reynell C, Attwell D (2011) The physiology of developmental changes in BOLD functional imaging signals. Dev Cogn Neurosci 1:199–216. 10.1016/j.dcn.2011.04.001 22436508 PMC6987565

[B22] Henson R (2004) Analysis of fMRI time series: linear time-invariant models, event-related fMRI, and optimal experimental design, Chap 40. In: Human Brain Function, Ed 2 (Frackowiak RSJ, Friston KJ, Frith CD, Dolan RJ, Price CJ, Zeki S, Ashburner JT, Penny WD, eds), pp 793–822. Burlington, MA: Academic.

[B23] Hu L, Valentini E, Zhang ZG, Liang M, Iannetti GD (2014) The primary somatosensory cortex contributes to the latest part of the cortical response elicited by nociceptive somatosensory stimuli in humans. Neuroimage 84:383–393. 10.1016/j.neuroimage.2013.08.057 24001456

[B24] Kozberg MG, Chen BR, DeLeo SE, Bouchard MB, Hillman EMC (2013) Resolving the transition from negative to positive blood oxygen level-dependent responses in the developing brain. Proc Natl Acad Sci U S A 110:4380–4385. 10.1073/pnas.1212785110 23426630 PMC3600493

[B25] Lloyd-Fox S, Blasi A, Volein A, Everdell N, Elwell CE, Johnson MH (2009) Social perception in infancy: a near infrared spectroscopy study. Child Dev 80:986–999. 10.1111/j.1467-8624.2009.01312.x 19630889

[B26] Lloyd-Fox S, Blasi A, Elwell CE (2010a) Illuminating the developing brain: the past, present and future of functional near infrared spectroscopy. Neurosci Biobehav Rev 34:269–284. 19632270 10.1016/j.neubiorev.2009.07.008

[B27] Lloyd-Fox S, Blasi A, Everdell N, Elwell CE, Johnson MH (2010b) Selective cortical mapping of biological motion processing in young infants. J Cogn Neurosci 23:2521–2532. 20954934 10.1162/jocn.2010.21598

[B28] Meek J (2002) Basic principles of optical imaging and application to the study of infant development. Dev Sci 5:371–380. 10.1111/1467-7687.00376

[B29] Meek J, Firbank M, Elwell CE, Atkinson J, Braddick O, Wyatt JS (1998) Regional hemodynamic responses to visual stimulation in awake infants. Pediatr Res 43:840–843. 10.1203/00006450-199806000-00019 9621996

[B30] Obrig H, Wenzel R, Kohl M, Horst S, Wobst P, Steinbrink J, Thomas F, Villringer A (2000) Near-infrared spectroscopy: does it function in functional activation studies of the adult brain? Int J Psychophysiol 35:125–142. 10.1016/S0167-8760(99)00048-3 10677642

[B31] Pillai Riddell R, Flora DB, Stevens SA, Stevens B, Cohen LL, Greenberg S, Garfield H (2013) Variability in infant acute pain responding meaningfully obscured by averaging pain responses. Pain 154:714–721. 10.1016/j.pain.2013.01.015 23531475

[B32] Roche-Labarbe N, Wallois F, Ponchel E, Kongolo G, Grebe R (2007) Coupled oxygenation oscillation measured by NIRS and intermittent cerebral activation on EEG in premature infants. Neuroimage 36:718–727. 10.1016/j.neuroimage.2007.04.002 17482837

[B33] Roche-Labarbe N, Zaaimi B, Berquin P, Nehlig A, Grebe R, Wallois F (2008) NIRS-measured oxy- and deoxyhemoglobin changes associated with EEG spike-and-wave discharges in children. Epilepsia 49:1871–1880. 10.1111/j.1528-1167.2008.01711.x 18631367

[B34] Schroeter ML, Kupka T, Mildner T, Uludağ K, von Cramon DY (2006) Investigating the post-stimulus undershoot of the BOLD signal—a simultaneous fMRI and fNIRS study. Neuroimage 30:349–358. 10.1016/j.neuroimage.2005.09.048 16257236

[B35] Slater R, Cantarella A, Gallella S, Worley A, Boyd S, Meek J, Fitzgerald M (2006) Cortical pain responses in human infants. J Neurosci 26:3662–3666. 10.1523/JNEUROSCI.0348-06.2006 16597720 PMC6674141

[B36] Slater R, Cantarella A, Yoxen J, Patten D, Potts H, Meek J, Fitzgerald M (2009) Latency to facial expression change following noxious stimulation in infants is dependent on postmenstrual age. Pain 146:177–182. 10.1016/j.pain.2009.07.022 19682794

[B37] Slater R, Worley A, Fabrizi L, Roberts S, Meek J, Boyd S, Fitzgerald M (2010a) Evoked potentials generated by noxious stimulation in the human infant brain. Eur J Pain 14:321–326. 19481484 10.1016/j.ejpain.2009.05.005

[B38] Slater R, Fabrizi L, Worley A, Meek J, Boyd S, Fitzgerald M (2010b) Premature infants display increased noxious-evoked neuronal activity in the brain compared to healthy age-matched term-born infants. Neuroimage 52:583–589. 20438855 10.1016/j.neuroimage.2010.04.253

[B39] Steffener J, Tabert M, Reuben A, Stern Y (2010) Investigating hemodynamic response variability at the group level using basis functions. Neuroimage 49:2113 10.1016/j.neuroimage.2009.11.014 19913625 PMC2818488

[B40] Steinbrink J, Villringer A, Kempf F, Haux D, Boden S, Obrig H (2006) Illuminating the BOLD signal: combined fMRI–fNIRS studies. Magn Reson Imaging 24:495–505. 10.1016/j.mri.2005.12.034 16677956

[B41] Taga G, Asakawa K, Maki A, Konishi Y, Koizumi H (2003) Brain imaging in awake infants by near-infrared optical topography. Proc Natl Acad Sci U S A 100:10722–10727. 10.1073/pnas.1932552100 12960368 PMC196871

[B42] Taylor KS, Davis KD (2009) Stability of tactile- and pain-related fMRI brain activations: an examination of threshold-dependent and threshold-independent methods. Hum Brain Mapp 30:1947–1962. 10.1002/hbm.20641 18711711 PMC6870907

[B43] Telkemeyer S, Rossi S, Koch SP, Nierhaus T, Steinbrink J, Poeppel D, Obrig H, Wartenburger I (2009) Sensitivity of newborn auditory cortex to the temporal structure of sounds. J Neurosci 29:14726–14733. 10.1523/JNEUROSCI.1246-09.2009 19940167 PMC6666009

[B44] Valentini E, Hu L, Chakrabarti B, Hu Y, Aglioti SM, Iannetti GD (2012) The primary somatosensory cortex largely contributes to the early part of the cortical response elicited by nociceptive stimuli. Neuroimage 59:1571–1581. 10.1016/j.neuroimage.2011.08.069 21906686

[B45] Vanhatalo S, Lauronen L (2006) Neonatal SEP—back to bedside with basic science. Semin Fetal Neonatal Med 11:464–470. 10.1016/j.siny.2006.07.009 16978936

[B46] Verriotis M, Fabrizi L, Lee A, Ledwidge S, Meek J, Fitzgerald M (2015) Cortical activity evoked by inoculation needle prick in infants up to one-year old. Pain 156:222–230. 10.1097/01.j.pain.0000460302.56325.0c 25599443 PMC4309489

[B47] Wallois F, Mahmoudzadeh M, Patil A, Grebe R (2012) Usefulness of simultaneous EEG–NIRS recording in language studies. Brain Lang 121:110–123. 10.1016/j.bandl.2011.03.010 21546072

[B48] Wartenburger I, Steinbrink J, Telkemeyer S, Friedrich M, Friederici AD, Obrig H (2007) The processing of prosody: evidence of interhemispheric specialization at the age of four. Neuroimage 34:416–425. 10.1016/j.neuroimage.2006.09.009 17056277

[B49] Woody CD (1967) Characterization of an adaptive filter for the analysis of variable latency neuroelectric signals. Med Biol Eng 5:539–554. 10.1007/BF02474247

[B50] Worley A, Fabrizi L, Boyd S, Slater R (2012) Multi-modal pain measurements in infants. J Neurosci Methods 205:252–257. 10.1016/j.jneumeth.2012.01.009 22285660 PMC3465552

[B51] Wyatt JS, Cope M, Delpy DT, van der Zee P, Arridge S, Edwards AD, Reynolds EO (1990) Measurement of optical path length for cerebral near-infrared spectroscopy in newborn infants. Dev Neurosci 12:140–144. 2335137 10.1159/000111843

[B52] Yücel MA, Aasted CM, Petkov MP, Borsook D, Boas DA, Becerra L (2015) Specificity of hemodynamic brain responses to painful stimuli: a functional near-infrared spectroscopy study. Sci Rep 5:9469. 10.1038/srep09469 25820289 PMC4377554

